# Fresh Produce as a Potential Vector and Reservoir for Human Bacterial Pathogens: Revealing the Ambiguity of Interaction and Transmission

**DOI:** 10.3390/microorganisms11030753

**Published:** 2023-03-15

**Authors:** Ahmed Esmael, Rashad R. Al-Hindi, Raed S. Albiheyri, Mona G. Alharbi, Amani A. R. Filimban, Mazen S. Alseghayer, Abdulaziz M. Almaneea, Meshari Ahmed Alhadlaq, Jumaa Ayubu, Addisu D. Teklemariam

**Affiliations:** 1 Botany and Microbiology Department, Faculty of Science, Benha University, Benha 13518, Egypt; 2 Nebraska Center for Virology, University of Nebraska-Lincoln, Lincoln, NE 68583, USA; 3 Department of Biological Sciences, Faculty of Science, King Abdulaziz University, Jeddah 21589, Saudi Arabia; 4 Monitoring and Risk Assessment Department, Saudi Food and Drug Authority, Riyadh 13513, Saudi Arabia; 5 Molecular Biology Section, Reference Laboratory for Microbiology Department, Research and Laboratories Sector, Saudi Food and Drug Authority, Riyadh 13513, Saudi Arabia

**Keywords:** fresh produce, foodborne bacteria, stomata, outbreak

## Abstract

The consumer demand for fresh produce (vegetables and fruits) has considerably increased since the 1980s for more nutritious foods and healthier life practices, particularly in developed countries. Currently, several foodborne outbreaks have been linked to fresh produce. The global rise in fresh produce associated with human infections may be due to the use of wastewater or any contaminated water for the cultivation of fruits and vegetables, the firm attachment of the foodborne pathogens on the plant surface, and the internalization of these agents deep inside the tissue of the plant, poor disinfection practices and human consumption of raw fresh produce. Several investigations have been established related to the human microbial pathogens (HMPs) interaction, their internalization, and survival on/within plant tissue. Previous studies have displayed that HMPs are comprised of several cellular constituents to attach and adapt to the plant’s intracellular niches. In addition, there are several plant-associated factors, such as surface morphology, nutrient content, and plant–HMP interactions, that determine the internalization and subsequent transmission to humans. Based on documented findings, the internalized HMPs are not susceptible to sanitation or decontaminants applied on the surface of the fresh produce. Therefore, the contamination of fresh produce by HMPs could pose significant food safety hazards. This review provides a comprehensive overview of the interaction between fresh produce and HMPs and reveals the ambiguity of interaction and transmission of the agents to humans.

## 1. Introduction

Fresh produce (vegetables and fruits) consumption has increased considerably since the 1980s because of the increasing consumer demand for a healthy life, particularly in developed countries. Following FAO guidelines, 400 g of fresh fruits and vegetables should be consumed daily [1]. Fresh produce diets have been shown to protect humans from some chronic ailments including cancer, diabetes, hypertension, obesity, and cardiovascular diseases [1].

Fresh produce is one of the crucial constituents of healthy food; nevertheless, they have been linked with several seasonal or global foodborne outbreaks, causing illnesses and serious economic losses. It has been estimated that nearly 76 million cases of foodborne diseases occur yearly in the United States [2]. Salmonella enterica (e.g., *S. enterica* serovar Typhimurium) and *Escherichia coli* (e.g., *E. coli* O157:H7) appear to be the most prevalent causative agents of foodborne infection linked to the ingestion of fresh produce [2]. These human pathogens are not known to be plant pathogens. Human microbial pathogens (HMPs) colonize and firmly attach to the plant surface or internalize into the plant tissues and sustain their population in the mesophyll without causing infection in the plant. Several laboratories’ microscopic-based investigations have shown the association of HMPs, particularly *E. coli* O157:H7 and *Salmonella* spp., with plant stomata, wounds, and lesions found on the leaf of the plant [3]. These HMPs are not easily removed or decontaminated with standard disinfection procedures [4].

During the last few years, the prevalence, incidence, severity, and spreading of human diseases associated with the ingestion of fresh green products have drawn the focus of farmers, food industry, consumers, researchers, and politicians [5]. According to the CDC report during the period between 1998 to 2013, 972 green raw products-associated outbreaks were reported causing 34,674 diseases, 2315 hospitalizations, and 72 mortalities in the U.S. [6]. Most of these diseases were caused by *E. coli* (10%), *Salmonella enterica* (21%), and norovirus (54% of outbreaks) [6]. This is attributed to the increased promotion and trend of consuming fresh green products. Lettuce (salad leaves) consumption has considerably increased (12.0 kg/person/year) in the U.S. during the past decade [7]. Additionally, in the U.S. the annual demand for packed salads has increased over the last two decades [8], which implies there was a real change in the consumers’ attitude towards buying slightly treated salads and/or ready-to-eat foods.

Fresh green products are vulnerable to pathogenic contamination during storage, production, packaging, processing, and transportation [9,10]. During the production of vegetables, the main vehicles for bacterial contamination are farm and municipal waste, manure soil amendments, irrigation water, and intrusion of wild animals [11,12,13]. For effective leafy green colonization, bacteria entail the capacity to adhere, internalize, and/or create biofilms to withstand exterior or interior disturbance and survive epiphytically. Both *E. coli* and *Salmonella* can modulate their cellular function upon the contact of leaf greens towards the generation of biomolecules that participated in attachment and biofilm formation [14]. Phylloplane settlement progressions are mostly accompanied by the internalization of the bacterial agent through the stomatal openings. Studies have displayed that the two most common leafy green contaminants, *E. coli* and *Salmonella*, can reach the intercellular regions of the leaf via the stomatal aperture [15,16,17,18]. Human bacteria could recognize plant cells through Microbe-Associated Molecular Patterns (MAMPs) to initiate defense responses associated with Pattern-Triggered Immunity (PTI) [19], including a diminution of the width of stomatal openings [18,20]. In contrast, bacteria could destabilize the stomatal closure defense to deal with such responses [18] or activate the expression of genes linked to antimicrobial resistance and oxidative stress tolerance [21].

The impact of plants and human bacterial pathogen interactions on the leaf is profoundly affected by agents’ persistence time in/on leafy greens [22,23]. The viability of bacterial pathogens in the phyllosphere is mostly reliant on the species of the plant and their genotypes [24,25,26,27,28,29,30,31]. Intra- and inter-specific variations of certain leafy traits have resulted in a variation in bacterial colonization. Research findings indicated that varying *E. coli* O157:H7 persistence on spinach leaves has been affected by the roughness of the leaf blade and the density of the stomata. Other factors associated with the surface of the leaf, including hydrophobicity, level of epicuticular wax, and vein density, were linked to cultivar-specific differences in *S. enterica* ser. Senftenberg attachment on Batavia type lettuces and iceberg [28]. In tomatoes, the genotype of the plant influenced *S. enterica* persistence in the phyllosphere after the dip-inoculation with a cocktail of eight-serovars (Mbandaka, Baildon, Cubana, Enteritidis, Newport, Havana, Schwarzengrund, and Poona) [24]. In addition, the colonization of lettuce and tomato seedlings by *S. enterica* could be affected by the plant species, cultivar, bacterial strains, and serovar [32]. In general, plant–HMPs interaction is a complex science that involves several factors from different perspectives.

Safe production methods and proper decontamination or disinfection procedures are critical steps in ensuring the food safety of ready-to-eat foods and fresh produces. Most of fresh produce is eaten raw or minimally processed and does not undergo a ‘lethal’ process treatment, such as cooking. In addition, disinfection and cleaning are very important processes during food processing and packaging to ensure hygienic products and food safety [33]. The efficacy of various disinfectants and sanitizing methods for reducing the burden of microbial populations on raw fruits and vegetables varies greatly. Differences in the characteristics of the surface of the fresh produce, type and physiological state of microbial cells, the method and procedure used for disinfection (e.g., temperature, contact time, pH, dosage, residual concentration, etc.), and environmental stress conditions interact to influence the activity of disinfectants and sanitizers [34]. Vigorously washing vegetables and fruits with clean water minimizes the number of microorganisms by 10–100-fold and is often as effective as treatment with 200 ppm chlorine. To date, several types of physical and chemical methods are used for the decontamination of fresh produce to prevent the infection of humans with pathogenic microorganisms [35]. Most of the commercial methods are based on chemical principles, including chlorine dioxide (ClO_2_), ozone (O_3_), peracetic acid, hydrogen peroxide (H_2_O_2_)*,* edible coatings, cold plasma, and so on [33]. Physical non-thermal decontamination methods are effective at sub-lethal temperatures, thus it minimize negative consequences on the nutritional value of food [36]. These include the application of power ultrasound, gamma irradiation, UV treatment, high hydrostatic pressure, beta irradiation, and pulsed light. They are efficient but applicable to certain types of food matrices and use more time and energy. Purely physical procedures, such as high hydrostatic pressure, are chemically secure, but they necessitate complicated and costly equipment [37], and this can affect the quality of food products [38].

Some research findings indicated that the application of physical or chemical methods fails in removing bacterial contamination from the surface of fresh produce. These phenomena are mainly associated with the internalization of the microbial agent deep inside the tissue of the plant as discussed above. Hence, a clear and comprehensive understanding of the biological and molecular interaction between HMPs and fresh produce and other associated factors are crucial to select the appropriate disinfectant method and very helpful for designing of new disinfection approach.

There is no comprehensive literature review summarizing the past and present research outputs related to the interaction between the HMPs and fresh produce. In this regard, this review discusses the interaction between fresh produce and human bacterial pathogens and related foodborne outbreaks. We reviewed the available knowledge on bacterial internalization techniques into the tissue of plants and factors that influence the overall process.

## 2. Review Methodology

**Figure 1:**
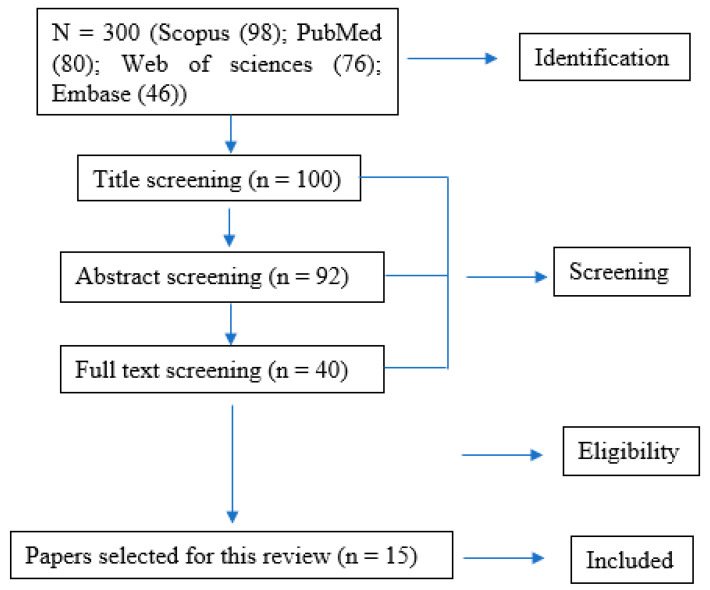
Schematic diagram showing the procedure of selection and screening of literature.

All published articles were searched in international databases, including Scopus, Medline (PubMed), Web of sciences, and Embase. The last search was done on 20 December 2022, and the English language used was while searching. The keyword search terms were a combination of the following: foodborne pathogens, green vegetables, fresh produce, pathogen internalization, reservoir, and vector. Additional searches were done for common foodborne bacteria, including *E. coli* O157:H7, *Salmonella*, and *Listeria monocytogenes*. The authors further narrowed the search for studies in bacteria internalization systems by searching for the names of specific types of systems, such as *stomata*, rhizosphere, plant tissue damage, and factors that determine the internalization process, such as biofilm, bacterial curli, flagella, cellulose, pili/fimbriae, plant surfaces, nutrient content, plant microbial flora, and foodborne outbreaks. For the present review, studies were excluded if they focused on plant bacterial pathogens or human pathogens other than bacterial pathogens. Based on these criteria, 15 papers were identified for discussion. In addition, the references of each article were reviewed to complement other studies (Figure 1). 

## 3. Bacteria-Fresh Produce Interaction (Bacterial Internalization Methods)

**Figure 2:**
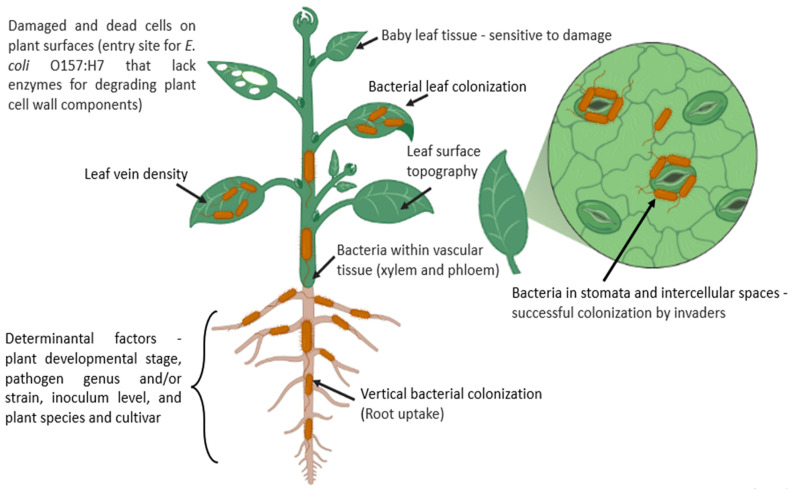
Schematic diagram illustrating the internalization of HMPs interior the plant tissue and determinants. Pathogens enter the plant tissue can be originated from the contaminated soil, animal manure, contaminated irrigation water, contaminated pesticides, and fertilizers. HMPs can be attracted to the rhizosphere and internalize into root tissues via root cracks and/or root-shoot transition areas (Vertical colonization). HMPS also enters the plant tissue via stomata and/or plant tissue damage.

HPMs internalization and attachment are multi-step procedures, which depend upon environmental, bacterial, and plant factors (Figure 2). HMPs’ entrance into plant tissues through natural openings (stomata, lenticels), roots, or wounds has been reported in several studies (Figure 2) [39]. Internalization facilitates HMPs to avoid adverse environmental conditions in plant tissue to get rid of UV and other environmental attacks and to get nutrients and water-rich niches inside the plants. Internalization could also shield HMPs from surface decontamination chemicals applied by consumers or companies [40].

### 3.1. Stomata

**Figure 3:**
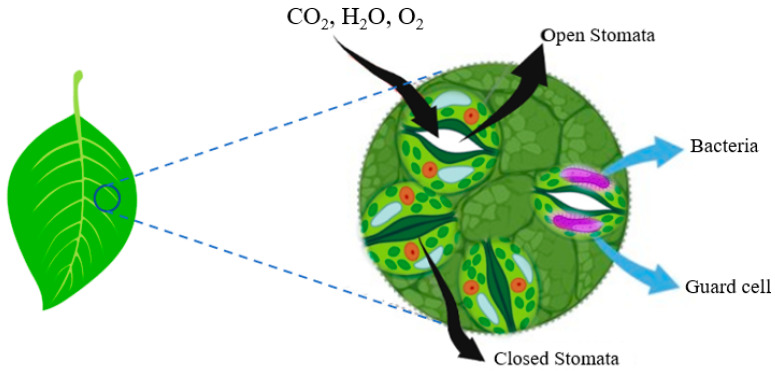
A schematic diagram showing the physiological function of plant stomata. It can serve as one route of entry for HMPs.

The stomatal pore is an abundant natural opening in the leaf epidermis [41] that helps in gas exchange essential for photosynthesis and is also a major route for bacteria internalized into the leaf interior (phloem, xylem, and intercellular space). Several electron microscopy studies revealed the interaction between HMPs on or near guard cells (Figure 3). For example, in a study conducted by Golberg and colleagues, *S. enterica* serovar Typhimurium SL1344 entered the iceberg and arugula lettuce through the stomata and localized in the sub-stomatal space [25]. In this study, despite the partial opening of stomata, no internalization of SL1344 was observed in parsley where most cells were found on the surface of the leaves [25]. Cells of *S. enterica* serovar Typhimurium MAE110, *E. coli* O157:H7 [17], and enteroaggregative *E. coli* [42] were linked to stomata in tomato, spinach, and Arugula leaves, respectively. Studies have shown that Shiga toxigenic *E. coli* (STEC) O157:H7 requires the type III secretion system (TTSS) to colonize stomata [17]. The STEC TTSS sec N mutants showed deficiency in the colonization of the plant stomata while K12 strains (non-pathogenic) containing a plasmid encoding the enterocyte effacement (LEE) pathogenicity island (effector genes and TTSS) were internalized more efficiently.

Stomata also serve as an active innate immunological agent against plants and HMP in Arabidopsis leaves [43]. According to previous studies, O157:H7 cannot withstand the closure of stomata, resulting in protracted stimulation of the stomatal immune response [43]. In contrast, a recent study found that S. enterica serovar Typhimurium SL1344 migrated toward stomata and internalized without stimulating an immune response [18]. This finding suggests that apart from plant pathogens [44], some HMPs have developed strategies to subvert the stomatal defense and were internalized into the plant tissue.

### 3.2. Rhizosphere or Root

The rhizosphere of plants is an environment that hosts a diverse group of microorganisms, including symbionts of plants and HMPs. Fresh produce plants’ root exudate is nutritionally rich and attracts *S. enterica* to the roots of lettuce plants [45]. Though HMPs cannot directly infiltrate via root cells, root cracks and lateral root emergence provide sites for the internalization of *S. enterica* and *E. coli* O157:H7 into root tissues [45,46], and in some cases between the epidermal cells [45]. A study has reported higher *S. enterica* colonization in the shoot-root transition area [45]. Once internalized, HMPs like the cases of *Salmonella enterica* can be found in the endodermis, parenchyma, vascular system, and pericycle of lettuce roots and barley’s inner root cortex [45]. Thorough investigations of *E. coli* O157:H7 localization in root tissues indicated its colonization in the cytoplasm, cell wall, and apoplast [47]. The translocation of HMPs from roots to the phyllosphere maybe depends on the flagellum [48] or presumably via the vasculature [49]. The mechanism for the migration of HMPs from root cortex to root vasculature through Casparian strips and endodermis, and from roots to phyllosphere through vascular system remains uninvestigated. Despite surface sterilization, pathogens were observed on lettuce leaves produced from hydroponic systems cultivated with water containing S Typhimurium or *E. coli* O157:H7 [50]. 

### 3.3. Plant Tissue Damage

Plants are usually exposed to different agents, such as environmental mechanical stresses, humans, and herbivores, that cause damage (wounding) and open the plant for HPMs to internalize the plant tissue. Wounding offers nutrients to HMPs and enables their entry into the tissue and consequent colonization [51]. For example, one study showed that wounding facilitated the quick perpetuation of *E. coli* O157:H7 on lettuce. Documented findings indicated that the concentration of *E. coli* O157:H7 enhanced by 4.5-fold, 4-fold, and 11-fold post-inoculation (4 h) on large pieces of mechanically cut lettuce leaves that were further bruised and shredded [52].

## 4. Factors Affecting the Interaction between Pathogenic Bacteria and Fresh Produce

### 4.1. Factor Associated with Bacteriological Agents

The interaction between HMPs and fresh produce depends on different factors, and one of these factors are linked with the pathogen by itself. HMPs population size [53], bacteria species or strain involved [54], and the presence of bacterial cell surface appendages like pili/fimbriae, curli, flagella, and cellulose. Bacterial biofilm is also another factor that determines the plant–pathogen interaction [14]. Some of these factors are discussed below.

#### 4.1.1. Biofilm

A biofilm is one of the most effective mechanisms used by HMPs to generate evasive fitness against immunologically challenging environments on or inside plants. Microbial biofilms can form on the surfaces of leaves and roots, as well as within plant tissues’ intercellular spaces. Biofilms protect bacteria from desiccation, UV radiation, environmental stress, and defense immunity of plants. They also protect against antimicrobial agents produced by normal flora or by the plant itself. A microbial biofilm also generates a protective coat against disinfectants and antiseptics used during food processing [55]. A biofilm is a mechanism by which HMPs survive in a nutrient-poor microenvironment inside or on the plant surface.

#### 4.1.2. Bacterial Curli

**Figure 4:**
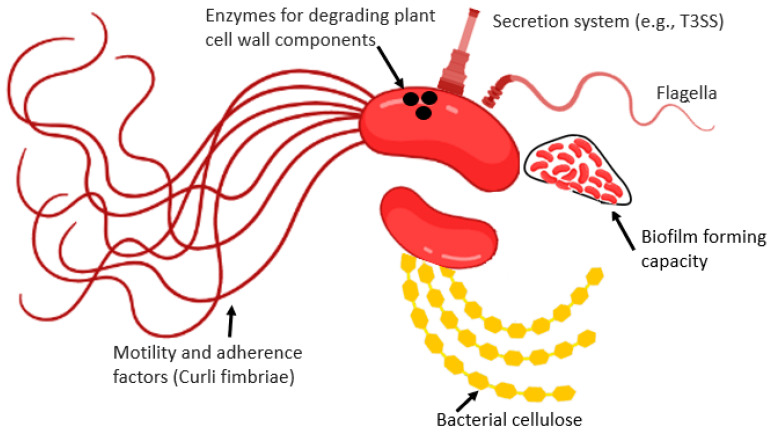
Some of the bacteriological factors which determine the internalization of HMPs inside the plant tissue.

Curli are the main proteinaceous constituent of extra-cellular matrix synthesized by many enterobacterial pathogens. Curli fibers participate in cell aggregation, attachment to the plant surfaces, and biofilm formation. Curli are also involved in host cell attachment and invasion, and they are crucial inducers of the plant immune response (Figure 4). A study by Macarisin et al. [56] indicated that curli-expressing *E. coli* O157:H7 strains developed stronger linkage with the fresh produce leaf surface, whereas curli-deficient mutants adhered to spinach at a significantly lower population [56].

#### 4.1.3. Flagella

Flagella are motility organelles, which facilitate reaching favorable habitats and serve as adhesive material to enhance their capability to attach to plant surfaces (Figure 4). The bacteriological agents adhere and irreversibly attach to the plant surface to develop microcolonies. They secrete EPS for the interactions between cells and plant surfaces. They also develop complex biofilm structures by interacting with alternative matrix components. The association of *S. enterica* internalization in leaves with chemotaxis and motility has been reported by Kroupitski et al. [16]. Motility-deficient flagella mutants (*fliGHI*::Tn10) were unable to properly attach and penetrate the lettuce leaves. It also restricted the *cheY* mutant defective entrance to chemotaxis.

#### 4.1.4. Cellulose and Pili/Fimbriae

The extracellular matrix, cellulose is crucial for the attachment of *Salmonella*. A lower level of colonization was noted in *bcsA* (cellulose synthase) lacking *S. enterica* Enteritidis mutant in alfalfa sprouts as compared to wild type. However, normal colonization capability was achieved after the plasmid-based *bcsA* expression [57]. 

Adhesins containing hair-like Pili/fimbriae (P, 1, F1C, and S in *E. coli*) are present on the bacterial cell surface that exhibit affinity to various carbohydrates. The interaction of adhesins with mammal components is either non-specific (electrostatic or hydrophobic) or specific (binding with specific host cell receptor moieties), which carries out tropism for the adhesion with specific tissue or host [58]. *Salmonella* and *E. coli* adhesins and fimbriae (amyloid curli fimbriae) have been studied concerning their plant adhesions. Curli is known to facilitate the *Salmonella* and *E. coli* attachments to leaves and sprouts, but their inactivation effect is low.

#### 4.1.5. Other Factors

Multiple studies have elaborated on the surface charge of bacterial cells, hydrophobicity, divalent cations, and capsule production during active or passive *E. coli* attachment to lettuce tissues [59]. These studies presented only a minor correlation between hydrophobicity, charge, cell surface appendages, and bacterial attachment capability with lettuce. Therefore, Span85 (hydrophobic surfactant) treatment could only detach 80% *E. coli* O157:H7 from lettuce leaves. The surfactant was also unable to detach pathogens from the cut edges, which indicates a heterogeneous surface nature [59]. Contrarily, a linear correlation between *Salmonella* cell surface hydrophobicity and its attachment capability to melon fruits has been reported [60].

### 4.2. Plant Factors

The colonization and interaction of foodborne pathogens (e.g., *Salmonella enterica* and *Escherichia coli*) with the plant immune system have been documented in various studies [19]. Plant factors include attachment sites [61], properties of plant surfaces [62], plant nutritive constituents and growing conditions [63,64], development stage [16,65], plant’s cultivar [24,26], and contamination site [66]. In some situations, like the case of STEC, the rate of internalization is dependent on multiple factors, including the plant species and tissue [67] and how plants are propagated [68]. 

#### 4.2.1. Properties of Plant Surfaces

Most of the aerial surfaces of the plants are covered with a hydrophobic cuticle that is mainly composed of polysaccharides, waxes, and fatty acids. It favors the attachment of hydrophobic molecules, whereas hydrophilic structures become exposed at the breaking points in the cuticle [62]. This situation helps the bacteria on the root surface to enter the plant cells generally covered with polysaccharides (pectin and cellulose) and glycoproteins. Such molecules are hydrophilic and can be negatively charged in some cases [69]. The attachment strength is correlated with the charge on the plant surface [60]. However, the exact binding sites or receptors remain unknown. The study of *S. Typhimurium’s* attachment to potato slices has revealed bacterial attachment to cell wall junctions. Bacteria were particularly noted to attach with the pectin layer at the cell wall junctions that could be the bacterial binding site [70]. Contrarily, another study has demonstrated a reduced *Salmonella* attachment to the components of the cell wall mainly containing pectin. Therefore, it could be deduced that pectin is less favorable for bacterial attachment than cellulose [71].

Plant surface architecture and topography are crucial for microbial adhesion. Similarly, roughness is also important for bacterial survival and adherence to plant tissues. *E. coli* O157:H7 adhesion to the leaves of various spinach cultivars has been investigated [27]. Plant leaves’ surface roughness depends on the leaf age and plant nature. During a study, high *Salmonella* affinity was noted for the old artificially contaminated leaves as compared to young lettuce leaves. A higher *S. Typhimurium* localization near the petiole has been noted. Similarly, a high bacterial affinity to the abaxial leaf side was observed as compared to the adaxial side [25]. Cantaloupe netting fissures are favorable *Salmonella* attachment sites, which help in their survival against sanitizers [72]. 

#### 4.2.2. Nutrient Content and Its Location in the Plant Tissue

Microflora distribution on the leaf surface is not homogenous and bacterial cells prefer to colonize at specific sites on the leaf surface, such as stomata, trichomes base, junctions of the epidermal cell wall, grooves or depressions near veins, and beneath the cuticle [73]. These points are rich in nutrients and water and protect bacteria from stress. Plant appendages (secretory ducts or cavities) could release metabolites. Glandular trichomes outgrow from the epidermis and act as an accumulation and secretion site for various compounds including defensive proteins, Pb ions, Ca, Mn, Na, and secondary metabolites (phenylpropanoids, monoterpenoids, and essential oils). Bacterial presence on the lower leaf surface is generally higher than on the upper surface. This might be due to low radiation, a thin layer of cuticle, and high trichomes and stomata density [61]. Therefore, the conditions are much better for the growth and survival of bacteria at the lower surface of the leaf as compared to other leaf parts.

Most human pathogenic bacterial strains, including STEC, preferentially colonize the roots and rhizosphere of fresh produce plants over leafy tissue and are internalized by plant tissue, where they can persist in the apoplastic space as an endophyte [66]. The apoplast contains metabolites, such as solutes, sugars, proteins, and cell wall components [66], and as such, it provides a rich environment for many bacterial species, including both commensal bacteria and human pathogens [66].

Similar behavior of human enteric pathogens has been documented on leaves with minor differences. *Salmonella enterica* serovar Thompson could attach in the cell margins and around the stomata of spinach leaves where the presence of native bacteria is detected [74]. The confocal microscopy of *E. coli* attachment at trichomes and stomata of cut lettuce plants revealed its attachment similarity with plant pathogens [15]. The stomata serve as protective bacterial niches and nutrient sources. Golberg and colleagues [25] confirmed the preference of this niche by *Salmonella* cells by demonstrating their high localization within and near lettuce leaves stomata. However, *Salmonella* colonization around stomata is limited to only a few serovars on specific plants. Contrarily, *E. coli* could better attach to cut lettuce surfaces, whereas *Pseudomonas fluorescens* prefers to attach on intact surfaces. However, *S. Typhimurium* could attach to both intact and cut surfaces [75]. The localization capability of enteric pathogens at similar leaf adhesion sites with plant pathogens and natural microflora helps in their long-term survival.

Enteric bacteria penetrate the soil through fertilizers, water, or directly through roots during hydroponic growth to attach to the host plant rhizosphere. Then, they invade and move to upper plant parts [76]. In contrast to fruits and leaves, the location of these bacterial attachments was significantly different than natural microflora. Natural plant pathogens and microflora generally attach to the trichome root hairs and epidermis. Plant pathogens could rapidly bind at the wound sites and cut ends of roots, whereas their binding at root tips is poor [77]. Contrarily, *E. coli* strains preferably attached at alfalfa sprouts root tip, but their attachment to the roots was quite slow. However, not all the studied *E. coli* strains bind to root hairs [78]. 

#### 4.2.3. Decontamination Methods Employed

*E. coli* and *Salmonella* attachment is considered an active step; however, this assumption is not supported by all the studies. In one study, for instance, only *Salmonella* viable cells could attach to potato slices [70]. Contrarily, the attachment levels of killed *E. coli* O157:H7, live *E. coli* O157:H7, and fluorescent polystyrene microspheres remained similar [79]. The differential results could be associated with the varying methods of bacterial inactivation. Glutaraldehyde was used to inactivate the *E. coli* cells, which could potentially change the bacterial adhesive features whereas various methods (thermal, ethanol, formalin, and kanamycin) were adopted to inactivate *Salmonella* cells [70]. Fresh produce-related pathogens are difficult to wash with antimicrobial and chlorine solutions [80]. Several studies have reported that chemicals-based washing of production-associated pathogens could be achieved from 1 (minimum) to 3 logs (maximum) [12,81]. Recently, less susceptibility to enteric pathogens has been reported against common sanitizers (chlorine) as compared to indigenous microorganisms. It suggests that the pathogens remaining after sanitizing could survive and grow on wet products with comparatively less competition [81].

#### 4.2.4. Plant Microbial Flora and Bacteria-to-Bacteria Interactions

**Figure 5:**
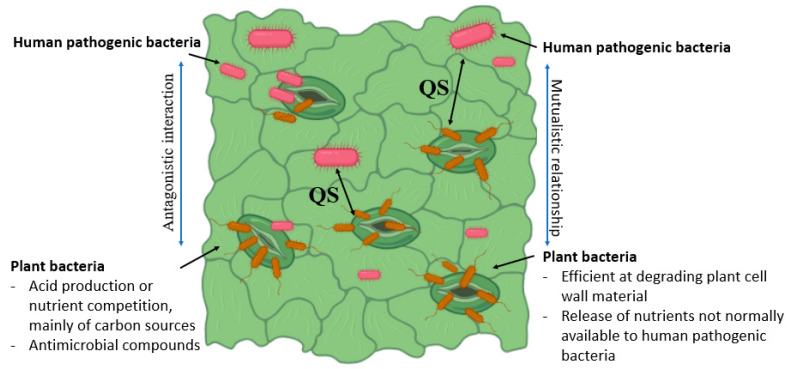
Schematic illustration of plant and HMPs intracellular interaction within the plant tissue. Bacterial communication occurs via small signaling molecules called quorum sensing (QS) factors, which are involved in the activation of virulence genes and the formation of biofilms and related immunological barriers.

Plant microbiota could inhibit or promote enteric pathogens’ establishment in plants (Figure 5). Plant diseases affect the phyllosphere atmosphere to promote the growth of the enteric pathogen. *Pectobacterium carotovorum* subsp. *Carotovorum* co-inoculation with *E. coli* O157:H7 or *S. enterica* increased their levels by more than 10-fold in comparison to individual inoculations [82]. Resident bacteria (*Erwinia herbicola* and *P. syringae*) and plant pathogens could enhance the *S. enterica* survival on leaves. An *S. enterica* viable population on plants pre-inoculated with one of two *E. herbicola* strains and *P. syringae* was increased by 10-fold compared to controls [83]. *Salmonella* protection from desiccation by plant epiphytic bacteria on leaf surfaces has been reported. Recently, Potnis et al. have revealed pathogen-associated molecular pattern (PAMP)-triggered immunity suppression by a virulent strain *X. perforans*. *X. perforans* generate an *S. enterica*-friendly environment by inducing effector-triggered susceptibility in tomato phyllosphere. However, the *S. enterica* population was reduced by an avirulent strain of *X. perforans*, which activated the effector-triggered immunity [84]. Several investigations have reported that the presence of other microbes helps in enteric pathogen colonization in leaf environment. However, a reduction in soft rot progression and *P. carotovorum* subsp. *carotovorum* population was noted in the presence of *S. enterica* (enteric pathogen), which moderated the pH of the local environment [85].

#### 4.2.5. Plant Immunity

Plants can avoid invading microbes through a complex innate immune system [53]. Plants generate a step-one response, which is triggered by molecules of conserved pathogens and degraded/modified plant products, which are referred to as pathogen or damage-associated molecular patterns (PAMP/DAMP). The conserved PAMPs are surface structure and cell wall components, which include lipopolysaccharides, chitin, and flagellin [86]. Plant extracellular pattern-recognition receptors (PRRs) could recognize PAMPs by passing intracellular signals to launch various defense molecules for restricting pathogenic invasion. Pathogen-triggered immunity (PTI) serves as the initial defense against infections [87].

The emerging field of human pathogens on plants (HPOP) has recently received the attention of phytopathologists and plant biologists. During the last decade, studies have focused on lipopolysaccharide (LPS), flagellin, and PAMPs to assess the human pathogen interaction with plants.

(i). LPS Perception

Plant and animal Gram-negative bacteria contain lipopolysaccharides (LPS) in the cell wall. An LPS is a well-characterized PAMP in animals, which is recognized through host Toll-like receptor 4 [88]. However, there are no known LPS-recognizing receptors in plants. Nonetheless, plants could perceive LPS derived from human pathogens leading to PTI activation. A significant stomatal closure in Arabidopsis has been reported in response to LPS purified from *E. coli* O55:B5, *S. Minnesota* R595, and *Pseudomonas aeruginosa* [20]. *Salmonella*-purified LPS is known to trigger extracellular alkalinization and ROS production in the cell suspension of tobacco [89]. However, it was unable to initiate these responses in tomato leaves [90], which suggests that LPS recognition might vary with the plant species and experimental conditions.

(ii). Flagellin Perception

Flagellin is a structural component of the bacterial flagellum that participates in bacterial motility and attachment on plants [48]. It induces plant immunity and is recognized through FLS2 receptor [19,90]. Similar to PTI elicitor flg22 [91], the flg22 epitope of *S. enterica* serovar Typhimurium 14,028 also serves as an efficient PAMP and elicitor of immune responses in tomato, tobacco, and Arabidopsis plants [90]. Flagellum-deficient *S. enterica* serovar Typhimurium 14,028 mutants colonize better on Arabidopsis, alfalfa, and wheat roots than wild-type bacterium [92]. It suggests *Salmonella* flagellum-based induction of plant defenses inhibit bacterial colonization on various organs.

### 4.3. Environmental Factors

The leaf environment is generally hostile to bacteria. The leaf surface faces rapid fluctuations in relative humidity, temperature, UV radiation, moisture (dew or rain), hydrophobicity, and nutrients [93]. Human and animal pathogens do not experience such significant fluctuations in a single day. Therefore, the survivability of these pathogens on the leaf surface environment could be questioned. However, the high occurrence of human pathogens (*E. coli* O157:H7 and *S. enterica*) in fresh produce (vegetables and sprouts) causing foodborne disease outbreaks reveals the fitness of human pathogens in the leaf environment.

## 5. Molecular Interactions

Recent studies have demonstrated the involvement of protein-encoding genes in the interaction between HMPs and their persistence in the rhizosphere and phyllosphere of leafy greens (Table 1). For instance, considerable attachment defects are not observed in flagella proteins and curli subunits encoding isogenic gene mutants [94,95]. These proteins participate in various attachment mediating pathways for the leafy green surface. These steps are performed by complex molecular connections involving single or multiple mechanisms according to the bacterial and plant genotypes. Stress response induction is crucial for pathogenic colonization and persistence under hostile plant environments. Oxidative (*oxyR*), nutrient limitation (*rpoS*) regulons, and cell envelope (*pspABC*) are some important stress responses in the plant environment [95,96]. These mechanisms facilitate human pathogens in adapting to reactive oxygen species, scarce nutrients, defense responses, and antimicrobial substances, which threaten their survival in plants [95,96]. The enteric bacteria conserve energy with better nutrient scavenging by altering their metabolism after stress responses.

## 6. Outbreaks Associated with Fresh Produce Consumption

The raw consumption of fresh produce without eliminating pathogens could lead to foodborne diseases [2]. *E. coli STEC* and *S. enterica* could cause fresh produce-related outbreaks of foodborne diseases (Table 2). Green leafy vegetables are the most common reservoirs for *E. coli* STEC infections including the “big six”. Contaminated water (drag water from cattle lots or water contaminated by other sources) is the most common source of contamination [100]. The *E. coli* O104:H4 outbreak in 2011 in North Germany is a recent example. A new strain of *E. coli* O104:H4 has caused the highest hemolytic uremic syndrome frequency and mortalities in a single outbreak. Imported fenugreek seeds from Egypt were most likely the outbreak source [101]. In the USA, 1779 foodborne outbreaks occurred from 2004 to 2010 with a confirmed etiology and food vehicle, and 9.2% (163) were fresh produce-related outbreaks [102]. An amount of 27.6% (45) of 163 produce-related outbreaks occurred in different U.S states. The same food contamination in various states is known as a multistate outbreak. From 2010 to 2017, 1797 foodborne outbreaks occurred in the U.S., where 12.7% (228) were related to fresh produce [102]. In 2020, a multistate outbreak of *E. coli* O157:H7 infections was associated with leafy greens, including romaine lettuce. A total of 40 individuals were infected across 19 states, leading to 10 reported hospitalizations and 4 cases of hemolytic uremic syndrome. Epidemiologic investigation identified leafy greens as the probable source of the outbreak; however, no specific type or brand of leafy greens was able to be identified. However, of 23 ill people interviewed, 22 reported eating leafy greens, wherein 15 consumed romaine.

## 7. Conclusions and Future Directions

Accumulating research findings indicate that fresh produce is one of the potential reservoirs of HMPs that cause disease in humans. Although there is no simple or single solution to this problem, one crucial aspect is to understand how human pathogens can penetrate and survive in plant tissues. This will lead to the discovery of a novel control approach and, consequently, reduce the rate of human infection.

As we have discussed in this review, several factors determine the survival of HMPs and their internalization (successful penetration) into the plant tissues. These factors could be either intrinsic (factors associated with the fresh produce) or extrinsic factors that are linked to the nature of the fitness of the HPMs, as well as the surrounding environment. Most of these factors positively or negatively contribute to the transmission of the pathogens to humans and determine the outcome or severity of the disease.

Some bacterial species may induce stronger immunity depending on how efficiently plants can recognize their system and trigger defense responses. Some bacteria may also evolve some unknown mechanisms to avoid recognition by the plant’s immune system and can penetrate and survive inside the plant tissue. Hence, a deep practical investigation is needed to broaden the understanding of plant-HMP interaction. In addition, like human pathogens, plant immune systems are diverse and need to be fully explored to understand the real interaction between them. This hidden mechanism may contribute to the internalization of foodborne human pathogens and the subsequent transmission of the agents to humans. Some plant pathogens or normal flora may support or suppress the activity of the human pathogenic bacteria, either inside or outside the tissue of the plant. Hence, this kind of interaction should be investigated at the molecular level.

In this review, we have demonstrated that the internalization of HMPs in plant tissues combined with their prolonged survival using nutrients and other essential substances creates a very serious public health risk. In the long run, this problem could be solved by developing novel physical and/or chemical disinfectants that can reach the target agent without damaging fresh produce’s nutritional value and the safety of humans. In addition, intense research is needed to know about the efficacy of currently available disinfectants and antiseptics items of the dose, concentration, duration of action, and other related factors that determine their efficacy. Homemade decontaminating agents should also be investigated for their efficacy in reducing the microorganisms found on the surface of the fresh produce and the efficacy of those approaches can be assessed in combination with commercially available disinfectants. Moreover, there are very limited research outputs related to the intercellular interaction between pathogens and the fresh produce. Hence, intensive research works are needed to explore the ambiguity of the interaction and its consequence on human health.

## Figures and Tables

**Table 1:** Major genes which are involved in the interaction between HMPs and green produce.

Bacterial Strains	Gene	Function	References
*Salmonella enterica* sv. Typhimurium	*flic*	Flagella biosynthesis	[42]
*E. coli* K-12	*fliN*	Flagella biosynthesis	[94]
*S. enterica* sv. Typhimurium	*bcsA*	Cellulose biosynthesis	[97]
*E. coli* K-12	*crl*	Regulation of curl formation	[94,95]
*E. coli* K-12	csgA	Curl formation and curl major subunit	[94,98]
*S. enterica* sv. Typhimurium	*yidR*	Putative ATP/GTP binding protein	[97]
*S. enterica* sv. Typhimurium	*misL*	Adhesin expressed from pathogenicity island-3	[97]
*E. coli* K-12, *E. coli* O157:H7	*ybiM*	Regulator of biofilm formation via the production of colonic acid	[95]
*S. enterica* sv. Typhimurium *S. enterica* sv. Saintpaul	*sirA*	Response regulator involved in biofilm formation	[99]
*S. enterica* sv. Typhimurium *S. enterica* sv. Saintpaul	*yigG*	Putative inner membrane protein of unknown function	[95]

**Table 2:** Major fresh produce outbreaks encountered globally (2013–2022).

Year	Country	Fresh Produce	Pathogen	Cases (Death)	References
2021	USA	leafy greens	*E. coli* O157:H7	40 (0)	[103]
2021	USA	Bright Farms Packaged Salad Greens	*Salmonella*	31 (0)	[104]
2018	USA	Chicken salad	*Salmonella* Typhimurium	265 (0)	[105]
2017	Canada	Romaine lettuce	*E. coli O157*	29 (0)	[106]
2016	UK	Salad mix	*E. coli O157*	161 (2)	[107]
2015–2016	United States and Canada	Packaged Leafy Green Salads	*Listeria monocytogenes*	16 (USA) 14 (Canada) (0)	[108]
2015	UK	Salad leaves	STEC O157:H7	51	[109]
2014	Norway	Salad mix	Yersinia enterocolitica O:9	0 (0)	[110]
2014	Norway	RTE salad mix	Salmonella enterica spp. enterica	0 (0)	[111]
2013	Sweden	Mixed green salad	*E. coli* O157:H7	19 (0)	[112]
2013	Canada	Lettuce (RTE)	*E. coli* O157:H7	31 (0)	[113]

## Data Availability

Not applicable.

## References

[1] (2021). The International Year of Fruits and Vegetables. https://www.fao.org/fruits-vegetables-2021/en/.

[2] Carstens C.K., Salazar J.K., Darkoh C. (2019). Multistate outbreaks of foodborne illness in the United States associated with fresh produce from 2010 to 2017. Front. Microbiol..

[3] de Oliveira Elias S., Noronha T.B., Tondo E.C. (2019). *Salmonella* spp. and *Escherichia coli* O157: H7 prevalence and levels on lettuce: A systematic review and meta-analysis. Food Microbiol..

[4] van Dijk H.F.G., Verbrugh H.A., Ad Hoc Advisory Committee on Disinfectants of the Health Council of the Netherlands (2022). Resisting disinfectants. Commun. Med..

[5] Rahman M., Alam M.-U., Luies S.K., Kamal A., Ferdous S., Lin A., Sharior F., Khan R., Rahman Z., Parvez S.M. (2022). Contamination of fresh produce with antibiotic-resistant bacteria and associated risks to human health: A scoping review. Int. J. Environ. Res. Public Health.

[6] Bennett S., Sodha S., Ayers T., Lynch M., Gould L., Tauxe R. (2018). Produce-associated foodborne disease outbreaks, USA, 1998–2013. Epidemiol. Infect..

[7] USDA Vegetables and Pulses Yearbook Tables. https://www.ers.usda.gov/data-products/vegetables-and-pulses-data/vegetables-and-pulses-yearbook-tables/.

[8] https://arefiles.ucdavis.edu/uploads/filer_public/fb/7b/fb7b6380-cdf9-4db5-b5d2-993640bcc1e6/freshcut2016cook20160926final.pdf.

[9] Barak J.D., Schroeder B.K. (2012). Interrelationships of food safety and plant pathology: The life cycle of human pathogens on plants. Annu. Rev. Phytopathol..

[10] Sapers G.M., Doyle M.P. (2014). Scope of the produce contamination problem. The Produce Contamination Problem.

[11] Jay-Russell M.T. (2014). What is the risk from wild animals in food-borne pathogen contamination of plants?. CABI Rev..

[12] Allende A., Monaghan J. (2015). Irrigation water quality for leafy crops: A perspective of risks and potential solutions. Int. J. Environ. Res. Public Health.

[13] Jiang X., Chen Z., Dharmasena M. (2015). The role of animal manure in the contamination of fresh food. Advances in Microbial Food Safety.

[14] Yaron S., Römling U. (2014). Biofilm formation by enteric pathogens and its role in plant colonization and persistence. Microb. Biotechnol..

[15] Seo K., Frank J. (1999). Attachment of *Escherichia coli* O157: H7 to lettuce leaf surface and bacterial viability in response to chlorine treatment as demonstrated by using confocal scanning laser microscopy. J. Food Prot..

[16] Kroupitski Y., Golberg D., Belausov E., Pinto R., Swartzberg D., Granot D., Sela S. (2009). Internalization of *Salmonella enterica* in leaves is induced by light and involves chemotaxis and penetration through open stomata. Appl. Environ. Microbiol..

[17] Saldaña Z., Sánchez E., Xicohtencatl-Cortes J., Puente J.L., Girón J.A. (2011). Surface structures involved in plant stomata and leaf colonization by Shiga-toxigenic *Escherichia coli* O157: H7. Front. Microbiol..

[18] Roy D., Panchal S., Rosa B.A., Melotto M. (2013). *Escherichia coli* O157: H7 induces stronger plant immunity than *Salmonella enterica* Typhimurium SL1344. Phytopathology.

[19] Garcia A.V., Charrier A., Schikora A., Bigeard J., Pateyron S., de Tauzia-Moreau M.-L., Evrard A., Mithöfer A., Martin-Magniette M.L., Virlogeux-Payant I. (2014). *Salmonella enterica* flagellin is recognized via FLS2 and activates PAMP-triggered immunity in Arabidopsis thaliana. Mol. Plant.

[20] Melotto M., Underwood W., Koczan J., Nomura K., He S.Y. (2006). Plant stomata function in innate immunity against bacterial invasion. Cell.

[21] Van der Linden I., Cottyn B., Uyttendaele M., Vlaemynck G., Heyndrickx M., Maes M., Holden N. (2016). Microarray-based screening of differentially expressed genes of *E. coli* O157: H7 Sakai during preharvest survival on butterhead lettuce. Agriculture.

[22] Fonseca J., Fallon S., Sanchez C., Nolte K. (2011). *Escherichia coli* survival in lettuce fields following its introduction through different irrigation systems. J. Appl. Microbiol..

[23] Kisluk G., Yaron S. (2012). Presence and persistence of *Salmonella enterica* serotype Typhimurium in the phyllosphere and rhizosphere of spray-irrigated parsley. Appl. Environ. Microbiol..

[24] Barak J.D., Kramer L.C., Hao L.-Y. (2011). Colonization of tomato plants by *Salmonella enterica* is cultivar dependent, and type 1 trichomes are preferred colonization sites. Appl. Environ. Microbiol..

[25] Golberg D., Kroupitski Y., Belausov E., Pinto R., Sela S. (2011). *Salmonella* Typhimurium internalization is variable in leafy vegetables and fresh herbs. Int. J. Food Microbiol..

[26] Quilliam R.S., Williams A.P., Jones D.L. (2012). Lettuce cultivar mediates both phyllosphere and rhizosphere activity of *Escherichia coli* O157: H7. PLoS ONE.

[27] Macarisin D., Patel J., Bauchan G., Giron J.A., Ravishankar S. (2013). Effect of spinach cultivar and bacterial adherence factors on survival of *Escherichia coli* O157: H7 on spinach leaves. J. Food Prot..

[28] Hunter P.J., Shaw R.K., Berger C.N., Frankel G., Pink D., Hand P. (2015). Older leaves of lettuce (*Lactuca* spp.) support higher levels of *Salmonella enterica* ser. Senftenberg attachment and show greater variation between plant accessions than do younger leaves. FEMS Microbiol. Lett..

[29] Crozier L., Hedley P.E., Morris J., Wagstaff C., Andrews S.C., Toth I., Jackson R.W., Holden N.J. (2016). Whole-transcriptome analysis of verocytotoxigenic *Escherichia coli* O157: H7 (Sakai) suggests plant-species-specific metabolic responses on exposure to spinach and lettuce extracts. Front. Microbiol..

[30] Erickson M.C., Liao J.-Y., Payton A.S., Cook P.W., Den Bakker H.C., Bautista J., Pérez J.C.D. (2018). Fate of enteric pathogens in different spinach cultivars cultivated in growth chamber and field systems. Food Qual. Saf..

[31] Roy D., Melotto M. (2019). Stomatal response and human pathogen persistence in leafy greens under preharvest and postharvest environmental conditions. Postharvest Biol. Technol..

[32] Wong C.W., Wang S., Levesque R.C., Goodridge L., Delaquis P. (2019). Fate of 43 *Salmonella* strains on lettuce and tomato seedlings. J. Food Prot..

[33] Deng L.-Z., Mujumdar A.S., Pan Z., Vidyarthi S.K., Xu J., Zielinska M., Xiao H.-W. (2020). Emerging chemical and physical disinfection technologies of fruits and vegetables: A comprehensive review. Crit. Rev. Food Sci. Nutr..

[34] Gil M.I., Selma M.V., López-Gálvez F., Allende A. (2009). Fresh-cut product sanitation and wash water disinfection: Problems and solutions. Int. J. Food Microbiol..

[35] Cui H., Ma C., Li C., Lin L. (2016). Enhancing the antibacterial activity of thyme oil against *Salmonella* on eggshell by plasma-assisted process. Food Control.

[36] Tiwari B.K., O’Donnell C.P., Brunton N.P., Cullen P.J. (2009). Degradation kinetics of tomato juice quality parameters by ozonation. Int. J. Food Sci. Technol..

[37] Rastogi N., Raghavarao K., Balasubramaniam V., Niranjan K., Knorr D. (2007). Opportunities and challenges in high pressure processing of foods. Crit. Rev. Food Sci. Nutr..

[38] Kruk Z.A., Yun H., Rutley D.L., Lee E.J., Kim Y.J., Jo C. (2011). The effect of high pressure on microbial population, meat quality and sensory characteristics of chicken breast fillet. Food Control.

[39] Deering A.J., Pruitt R.E., Mauer L.J., Reuhs B.L. (2011). Identification of the cellular location of internalized *Escherichia coli* O157: H7 in mung bean, Vigna radiata, by immunocytochemical techniques. J. Food Prot..

[40] Niemira B.A., Cooke P.H. (2010). *Escherichia coli* O157: H7 biofilm formation on Romaine lettuce and spinach leaf surfaces reduces efficacy of irradiation and sodium hypochlorite washes. J. Food Sci..

[41] Bertolino L.T., Caine R.S., Gray J.E. (2019). Impact of stomatal density and morphology on water-use efficiency in a changing world. Front. Plant Sci..

[42] Berger C.N., Shaw R.K., Brown D.J., Mather H., Clare S., Dougan G., Pallen M.J., Frankel G. (2009). Interaction of *Salmonella enterica* with basil and other salad leaves. ISME J..

[43] Wang Z., Gou X. (2021). The first line of defense: Receptor-like protein kinase-mediated stomatal immunity. Int. J. Mol. Sci..

[44] Riggio G.M., Jones S.L., Gibson K.E. (2019). Risk of human pathogen internalization in leafy vegetables during lab-scale hydroponic cultivation. Horticulturae.

[45] Klerks M., Van Gent-Pelzer M., Franz E., Zijlstra C., Van Bruggen A. (2007). Physiological and molecular responses of *Lactuca* sativa to colonization by *Salmonella enterica* serovar Dublin. Appl. Environ. Microbiol..

[46] Tyler H.L., Triplett E.W. (2008). Plants as a habitat for beneficial and/or human pathogenic bacteria. Annu. Rev. Phytopathol..

[47] Wright K.M., Chapman S., McGeachy K., Humphris S., Campbell E., Toth I.K., Holden N.J. (2013). The endophytic lifestyle of *Escherichia coli* O157: H7: Quantification and internal localization in roots. Phytopathology.

[48] Cooley M.B., Miller W.G., Mandrell R.E. (2003). Colonization of Arabidopsis thaliana with *Salmonella enterica* and enterohemorrhagic *Escherichia coli* O157: H7 and competition by Enterobacter asburiae. Appl. Environ. Microbiol..

[49] Solomon E.B., Yaron S., Matthews K.R. (2002). Transmission of *Escherichia coli* O157: H7 from contaminated manure and irrigation water to lettuce plant tissue and its subsequent internalization. Appl. Environ. Microbiol..

[50] Franz E., Visser A.A., Van Diepeningen A.D., Klerks M.M., Termorshuizen A.J., van Bruggen A.H. (2007). Quantification of contamination of lettuce by GFP-expressing *Escherichia coli* O157: H7 and *Salmonella enterica* serovar Typhimurium. Food Microbiol..

[51] Savatin D.V., Gramegna G., Modesti V., Cervone F. (2014). Wounding in the plant tissue: The defense of a dangerous passage. Front. Plant Sci..

[52] Brandl M. (2008). Plant lesions promote the rapid multiplication of *Escherichia coli* O157: H7 on postharvest lettuce. Appl. Environ. Microbiol..

[53] Jacob C., Melotto M. (2020). Human pathogen colonization of lettuce dependent upon plant genotype and defense response activation. Front. Plant Sci..

[54] Kljujev I., Raicevic V., Jovicic-Petrovic J., Vujovic B., Mirkovic M., Rothballer M. (2018). Listeria monocytogenes—Danger for health safety vegetable production. Microb. Pathog..

[55] Carrascosa C., Raheem D., Ramos F., Saraiva A., Raposo A. (2021). Microbial biofilms in the food industry—A comprehensive review. Int. J. Environ. Res. Public Health.

[56] Macarisin D., Patel J., Bauchan G., Giron J.A., Sharma V.K. (2012). Role of curli and cellulose expression in adherence of *Escherichia coli* O157: H7 to spinach leaves. Foodborne Pathog. Dis..

[57] Barak J.D., Jahn C.E., Gibson D.L., Charkowski A.O. (2007). The role of cellulose and O-antigen capsule in the colonization of plants by *Salmonella enterica*. Mol. Plant-Microbe Interact..

[58] Wagner C., Hensel M. (2011). Adhesive mechanisms of *Salmonella enterica*. Bact. Adhes. Chem. Biol. Phys..

[59] Hassan A., Frank J. (2003). Influence of surfactant hydrophobicity on the detachment of *Escherichia coli* O157: H7 from lettuce. Int. J. Food Microbiol..

[60] Ukuku D.O., Fett W.F. (2006). Effects of cell surface charge and hydrophobicity on attachment of 16 *Salmonella* serovars to cantaloupe rind and decontamination with sanitizers. J. Food Prot..

[61] Karamanoli K., Thalassinos G., Karpouzas D., Bosabalidis A., Vokou D., Constantinidou H.-I. (2012). Are leaf glandular trichomes of oregano hospitable habitats for bacterial growth?. J. Chem. Ecol..

[62] Patel J., Sharma M. (2010). Differences in attachment of *Salmonella enterica* serovars to cabbage and lettuce leaves. Int. J. Food Microbiol..

[63] Ge C., Lee C., Lee J. (2012). The impact of extreme weather events on *Salmonella* internalization in lettuce and green onion. Food Res. Int..

[64] López-Gálvez F., Gil M.I., Allende A. (2018). Impact of relative humidity, inoculum carrier and size, and native microbiota on *Salmonella* ser. Typhimurium survival in baby lettuce. Food Microbiol..

[65] Pu S., Beaulieu J.C., Prinyawiwatkul W., Ge B. (2009). Effects of plant maturity and growth media bacterial inoculum level on the surface contamination and internalization of *Escherichia coli* O157: H7 in growing spinach leaves. J. Food Prot..

[66] Hirneisen K.A., Sharma M., Kniel K.E. (2012). Human enteric pathogen internalization by root uptake into food crops. Foodborne Pathog. Dis..

[67] Wright K.M., Crozier L., Marshall J., Merget B., Holmes A., Holden N.J. (2017). Differences in internalization and growth of *Escherichia coli* O157: H7 within the apoplast of edible plants, spinach and lettuce, compared with the model species *Nicotiana benthamiana*. Microb. Biotechnol..

[68] Erickson M.C., Webb C.C., Davey L.E., Payton A.S., Flitcroft I.D., Doyle M.P. (2014). Biotic and abiotic variables affecting internalization and fate of *Escherichia coli* O157: H7 isolates in leafy green roots. J. Food Prot..

[69] Torres A.G., Jeter C., Langley W., Matthysse A.G. (2005). Differential binding of *Escherichia coli* O157: H7 to alfalfa, human epithelial cells, and plastic is mediated by a variety of surface structures. Appl. Environ. Microbiol..

[70] Saggers E., Waspe C., Parker M., Waldron K., Brocklehurst T. (2008). *Salmonella* must be viable in order to attach to the surface of prepared vegetable tissues. J. Appl. Microbiol..

[71] Tan M.S., Rahman S., Dykes G.A. (2016). Pectin and xyloglucan influence the attachment of *Salmonella enterica* and Listeria monocytogenes to bacterial cellulose-derived plant cell wall models. Appl. Environ. Microbiol..

[72] Annous B.A., Solomon E.B., Cooke P.H., Burke A. (2005). Biofilm formation by *Salmonella* spp. on cantaloupe melons. J. Food Saf..

[73] Beattie G.A., Lindow S.E. (1999). Bacterial colonization of leaves: A spectrum of strategies. Phytopathology.

[74] Warner J., Rothwell S., Keevil C. (2008). Use of episcopic differential interference contrast microscopy to identify bacterial biofilms on salad leaves and track colonization by *Salmonella* Thompson. Environ. Microbiol..

[75] Takeuchi K., Matute C.M., Hassan A.N., Frank J.F. (2000). Comparison of the attachment of *Escherichia coli* O157: H7, Listeria monocytogenes, *Salmonella* Typhimurium, and Pseudomonas fluorescens to lettuce leaves. J. Food Prot..

[76] Lapidot A., Yaron S. (2009). Transfer of *Salmonella enterica* serovar Typhimurium from contaminated irrigation water to parsley is dependent on curli and cellulose, the biofilm matrix components. J. Food Prot..

[77] Matthysse A.G., Kijne J.W. (1998). Attachment of Rhizobiaceae to plant cells. The Rhizobiaceae: Molecular Biology of Model Plant-Associated Bacteria.

[78] Jeter C., Matthysse A.G. (2005). Characterization of the binding of diarrheagenic strains of *E. coli* to plant surfaces and the role of curli in the interaction of the bacteria with alfalfa sprouts. Mol. Plant-Microbe Interact..

[79] Solomon E., Matthews K. (2006). Interaction of live and dead *Escherichia coli* O157: H7 and fluorescent microspheres with lettuce tissue suggests bacterial processes do not mediate adherence. Lett. Appl. Microbiol..

[80] Kondo N., Murata M., Isshiki K. (2006). Efficiency of sodium hypochlorite, fumaric acid, and mild heat in killing native microflora and *Escherichia coli* O157: H7, *Salmonella* Typhimurium DT104, and Staphylococcus aureus attached to fresh-cut lettuce. J. Food Prot..

[81] Shirron N., Kisluk G., Zelikovich Y., Eivin I., Shimoni E., Yaron S. (2009). A comparative study assaying commonly used sanitizers for antimicrobial activity against indicator bacteria and a *Salmonella* Typhimurium strain on fresh produce. J. Food Prot..

[82] Wells J., Butterfield J. (1997). *Salmonella* contamination associated with bacterial soft rot of fresh fruits and vegetables in the marketplace. Plant Dis..

[83] Poza-Carrion C., Suslow T., Lindow S. (2013). Resident bacteria on leaves enhance survival of immigrant cells of *Salmonella enterica*. Phytopathology.

[84] Potnis N., Soto-Arias J.P., Cowles K.N., van Bruggen A.H., Jones J.B., Barak J.D. (2014). Xanthomonas perforans colonization influences *Salmonella enterica* in the tomato phyllosphere. Appl. Environ. Microbiol..

[85] Kwan G., Charkowski A.O., Barak J.D. (2013). *Salmonella enterica* suppresses *Pectobacterium carotovorum* subsp. *carotovorum* population and soft rot progression by acidifying the microaerophilic environment. MBio.

[86] Albert I., Hua C., Nürnberger T., Pruitt R.N., Zhang L. (2020). Surface sensor systems in plant immunity. Plant Physiol..

[87] Naveed Z.A., Wei X., Chen J., Mubeen H., Ali G.S. (2020). The PTI to ETI continuum in Phytophthora-plant interactions. Front. Plant Sci..

[88] De Jong H.K., Parry C.M., van der Poll T., Wiersinga W.J. (2012). Host—Pathogen interaction in invasive salmonellosis. PLOS Pathog..

[89] Shirron N., Yaron S. (2011). Active suppression of early immune response in tobacco by the human pathogen *Salmonella* Typhimurium. PLoS ONE.

[90] Meng F., Altier C., Martin G.B. (2013). S almonella colonization activates the plant immune system and benefits from association with plant pathogenic bacteria. Environ. Microbiol..

[91] Felix G., Duran J.D., Volko S., Boller T. (1999). Plants have a sensitive perception system for the most conserved domain of bacterial flagellin. Plant J..

[92] Iniguez A.L., Dong Y., Carter H.D., Ahmer B.M., Stone J.M., Triplett E.W. (2005). Regulation of enteric endophytic bacterial colonization by plant defenses. Mol. Plant-Microbe Interact..

[93] Driesen E., Van den Ende W., De Proft M., Saeys W. (2020). Influence of environmental factors light, CO_2_, temperature, and relative humidity on stomatal opening and development: A review. Agronomy.

[94] Hou Z., Fink R., Black E., Sugawara M., Zhang Z., Diez-Gonzalez F., Sadowsky M. (2012). Gene expression profiling of *Escherichia coli* in response to interactions with the lettuce rhizosphere. J. Appl. Microbiol..

[95] Fink R.C., Black E.P., Hou Z., Sugawara M., Sadowsky M.J., Diez-Gonzalez F. (2012). Transcriptional responses of *Escherichia coli* K-12 and O157: H7 associated with lettuce leaves. Appl. Environ. Microbiol..

[96] Kyle J.L., Parker C.T., Goudeau D., Brandl M.T. (2010). Transcriptome analysis of *Escherichia coli* O157: H7 exposed to lysates of lettuce leaves. Appl. Environ. Microbiol..

[97] Kroupitski Y., Brandl M., Pinto R., Belausov E., Tamir-Ariel D., Burdman S., Sela S. (2013). Identification of *Salmonella enterica* genes with a role in persistence on lettuce leaves during cold storage by recombinase-based in vivo expression technology. Phytopathology.

[98] Erickson M.C., Liao J., Payton A.S., Riley D.G., Webb C.C., Davey L.E., Kimbrel S., Ma L., Zhang G., Flitcroft I. (2010). Preharvest internalization of *Escherichia coli* O157: H7 into lettuce leaves, as affected by insect and physical damage. J. Food Prot..

[99] Salazar J.K., Deng K., Tortorello M.L., Brandl M.T., Wang H., Zhang W. (2013). Genes ycfR, sirA and yigG contribute to the surface attachment of *Salmonella enterica* Typhimurium and Saintpaul to fresh produce. PLoS ONE.

[100] Alharbi M.G., Al-Hindi R.R., Esmael A., Alotibi I.A., Azhari S.A., Alseghayer M.S., Teklemariam A.D. (2022). The “big six”: Hidden emerging foodborne bacterial pathogens. Trop. Med. Infect. Dis..

[101] Mariani-Kurkdjian P., Bingen E. (2012). *Escherichia coli* O104: H4: Un pathotype hybride. Arch. Pédiatrie.

[102] CDC National Outbreak Reporting System (NORS). https://wwwn.cdc.gov/norsdashboard/.

[103] CDC Outbreak of *E. coli* Infections Linked to Leafy Greens. https://col.st/BLGLr.

[104] CDC (2021). *Salmonella* Outbreak Linked to BrightFarms Packaged Salad Greens. Cent. Dis. Control. Prev..

[105] CDC Multistate Outbreak of Shiga Toxin-Producing Escherichia coli O157:H7 Infections Linked to Leafy Greens (Final Update). https://www.cdc.gov/ecoli/2017/o157h7-12-17/index.html.

[106] Agency C.F.I. Canadian Food Inspection Agency’s (CFIA) Investigation into *E. coli* O121 in Flour and Flour Products. https://inspection.canada.ca/about-cfia/transparency/regulatory-transparency-and-openness/food-safety-investigations/e-coli-o121/eng/1492621159359/1492621214587.

[107] CDC Multistate Outbreak of Shiga Toxin-Producing *Escherichia coli* Infections Linked to Flour (Final Update). https://www.cdc.gov/ecoli/2016/o121-06-16/.

[108] Self J.L., Conrad A., Stroika S., Jackson A., Whitlock L., Jackson K.A., Beal J., Wellman A., Fatica M.K., Bidol S. (2019). Multistate outbreak of listeriosis associated with packaged leafy green salads, United States and Canada, 2015–2016. Emerg. Infect. Dis..

[109] Mikhail A., Jenkins C., Dallman T., Inns T., Douglas A., Martín A., Fox A., Cleary P., Elson R., Hawker J. (2018). An outbreak of Shiga toxin-producing *Escherichia coli* O157: H7 associated with contaminated salad leaves: Epidemiological, genomic and food trace back investigations. Epidemiol. Infect..

[110] MacDonald E., Einöder-Moreno M., Borgen K., Thorstensen Brandal L., Diab L., Fossli Ø., Guzman Herrador B., Hassan A.A., Johannessen G.S., Johansen E.J. (2016). National outbreak of Yersinia enterocolitica infections in military and civilian populations associated with consumption of mixed salad, Norway, 2014. Eurosurveillance.

[111] Vestrheim D., Lange H., Nygård K., Borgen K., Wester A., Kvarme M., Vold L. (2016). Are ready-to-eat salads ready to eat? An outbreak of *Salmonella* Coeln linked to imported, mixed, pre-washed and bagged salad, Norway, November 2013. Epidemiol. Infect..

[112] Edelstein M., Sundborger C., Hergens M.-P., Ivarsson S., Dryselius R., Insulander M., Jernberg C., Hutin Y., Wallensten A. (2014). Barriers to trace-back in a salad-associated EHEC outbreak, Sweden, June 2013. PLoS Curr..

[113] Tataryn J., Morton V., Cutler J., McDonald L., Whitfield Y., Billard B., Gad R., Hexemer A. (2014). Foodborne illness and more: Outbreak of *E. coli* O157: H7 associated with lettuce served at fast food chains in the Maritimes and Ontario, Canada, Dec 2012. Can. Commun. Dis. Rep..

